# Primary Rotational Stability of Various Megaprostheses in a Biomechanical Sawbone Model with Proximal Femoral Defects Extending to the Isthmus

**DOI:** 10.1371/journal.pone.0129149

**Published:** 2015-06-01

**Authors:** Stefan Kinkel, Jan Nadorf, Jan Dennis Graage, Eike Jakubowitz, Jan Philippe Kretzer

**Affiliations:** 1 Laboratory of Biomechanics and Implant Research, Clinic for Orthopedics and Trauma Surgery, Center for Orthopedics, Trauma Surgery and Spinal Cord Injury, Heidelberg University Hospital, Heidelberg, Germany; 2 Laboratory for Biomechanics and Biomaterials, Department of Orthopaedic Surgery, Hannover Medical School, Hannover, Germany; Université de Technologie de Compiègne, FRANCE

## Abstract

**Purpose:**

Fixation of proximal femoral megaprostheses is achieved in the diaphyseal isthmus. We hypothesized that after extended bone resection including the proximal part of the isthmus a reduced length of fixation will affect the stability and fixation characteristics of these megaprostheses. The aim of this study was to analyze in a validated sawbone model with extended proximal femoral defects which types of implants have sufficient primary stability to allow osteointegration and to describe their fixation characteristics.

**Methods:**

Four different cementless megaprostheses were implanted into 16 Sawbones with an AAOS type III defect after resection 11cm below the lesser trochanter involving the proximal isthmus. To determine the primary implant stability relative micromotions between bone and implant were measured in relation to a cyclic torque of 7Nm applied on the longitudinal axis of the implant. We determined the fixation characteristics of the different implant designs by comparing these relative micromotions along the longitudinal stem axis.

**Results:**

In the tested sawbones all studied implants showed sufficient primary stability to admit bone integration with relative micromotions below 150µm after adapting our results to physiologic hip joint loadings. Different fixation characteristics of the megaprostheses were determined, which could be explained by their differing design and fixation concepts.

**Conclusions:**

Cementless megaprostheses of different designs seem to provide sufficient primary stability to bridge proximal femoral defects if the diaphyseal isthmus is partially preserved. In our sawbone model the different implant fixation patterns can be related to their stem designs. No evidence can be provided to favor one of the studied implants in this setting. However, femoral morphology is variable and in different isthmus configurations specific implant designs might be appropriate to achieve the most favorable primary stability, which enables bone integration and consequently long term implant stability.

## Introduction

Implantation of megaprostheses after resection of bone tumors allows for reconstruction of the bone defect [1]. Cementless fixation of these implants in the femoral diaphysis of the predominantly younger patients is commonly intended, as this seems to correlate with lower rates of aseptic loosening [2–4]. Loosening constitutes a decrease in secondary implant stability, which develops after bone ingrowth into the fixating parts of cementless prostheses. As formation of secondary stability is based on the initial press-fit fixation of the implant, so-called primary stability [5], a correlation between this factor for early bone integration and aseptic loosening exists [6, 7].

To achieve an adequate primary stability with press-fit, cementless stems rely on tight contact to the cortical bone. Interaction between implant design and the shape of the femoral canal determine this contact. The tightest area of the femur, which is the isthmus, might therefore be a crucial factor in implant stability. Depending on the extent of bone resection, this portion of the femur can be affected. Consequently the reduction of the femoral isthmus leads to different fixation conditions of cementless megaprostheses.

**Fig 1:**
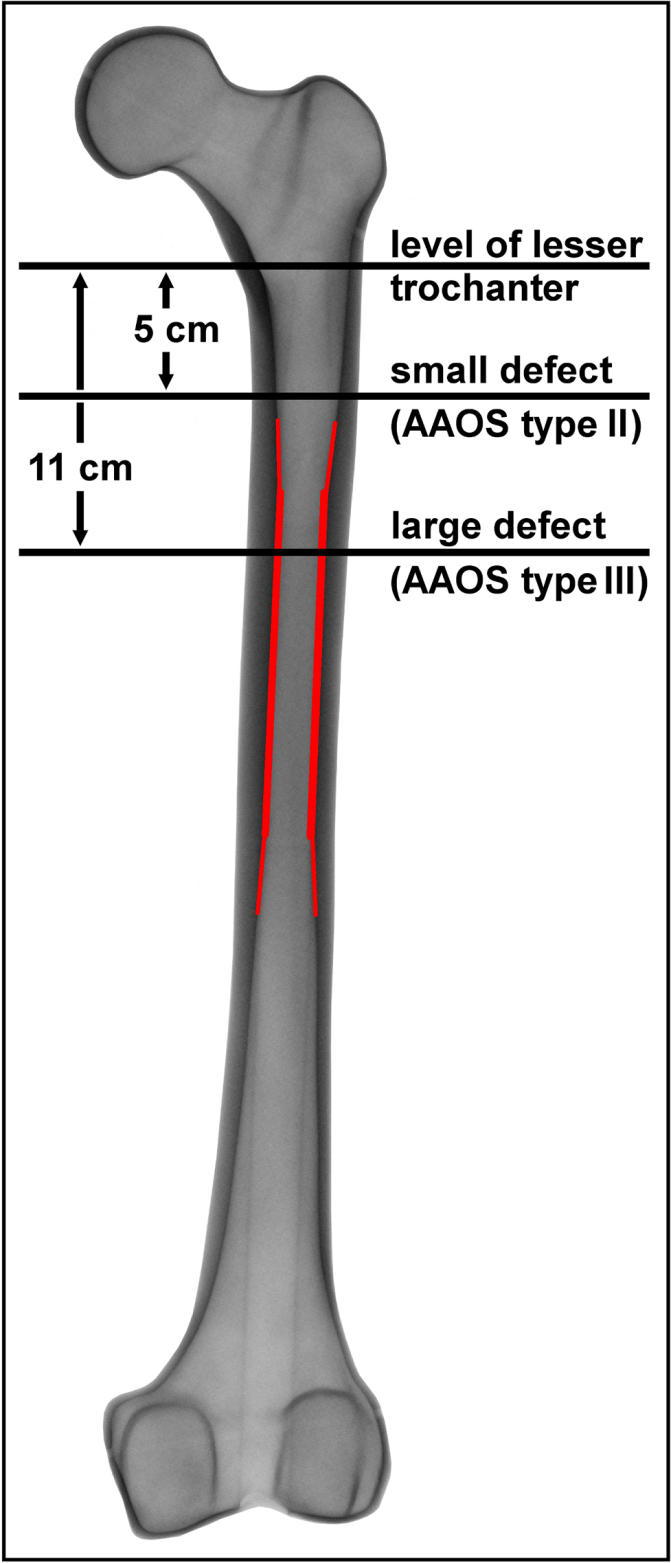
Used segmental AAOS defects of the proximal femur. The femoral isthmus region has been colored.

In a previous study of primary stability of megaprostheses of the proximal femur [8] we could show that adequate fixation conditions were achieved if the entire femoral isthmus was intact (AAOS II bone defect [9], Fig 1). However, there is no data describing the fixation properties of cementless megaprostheses in bone defects affecting the femoral isthmus. Therefore, the aim of this study was to evaluate the influence of bone defect extension to the isthmus on the fixation of different cementless megaprostheses. Thus, in an AAOS III bone defect [9], providing only the distal part of femoral isthmus (Fig 1), primary stability of potentially different fixating stems in comparison to each other as well as to their stability in the AAOS type II bone defect [8] should be studied. We hypothesized that in extended proximal defects (AAOS III bone defect) differences in stem design would result in a difference in primary rotational stability. Because the proximal part of the isthmus is absent and the stems are consequently implanted in the femur more distally with widening cortices below the isthmus, we expected compromised primary stability in stems relying on more distal fixation.

## Material and Methods

**Fig 2:**
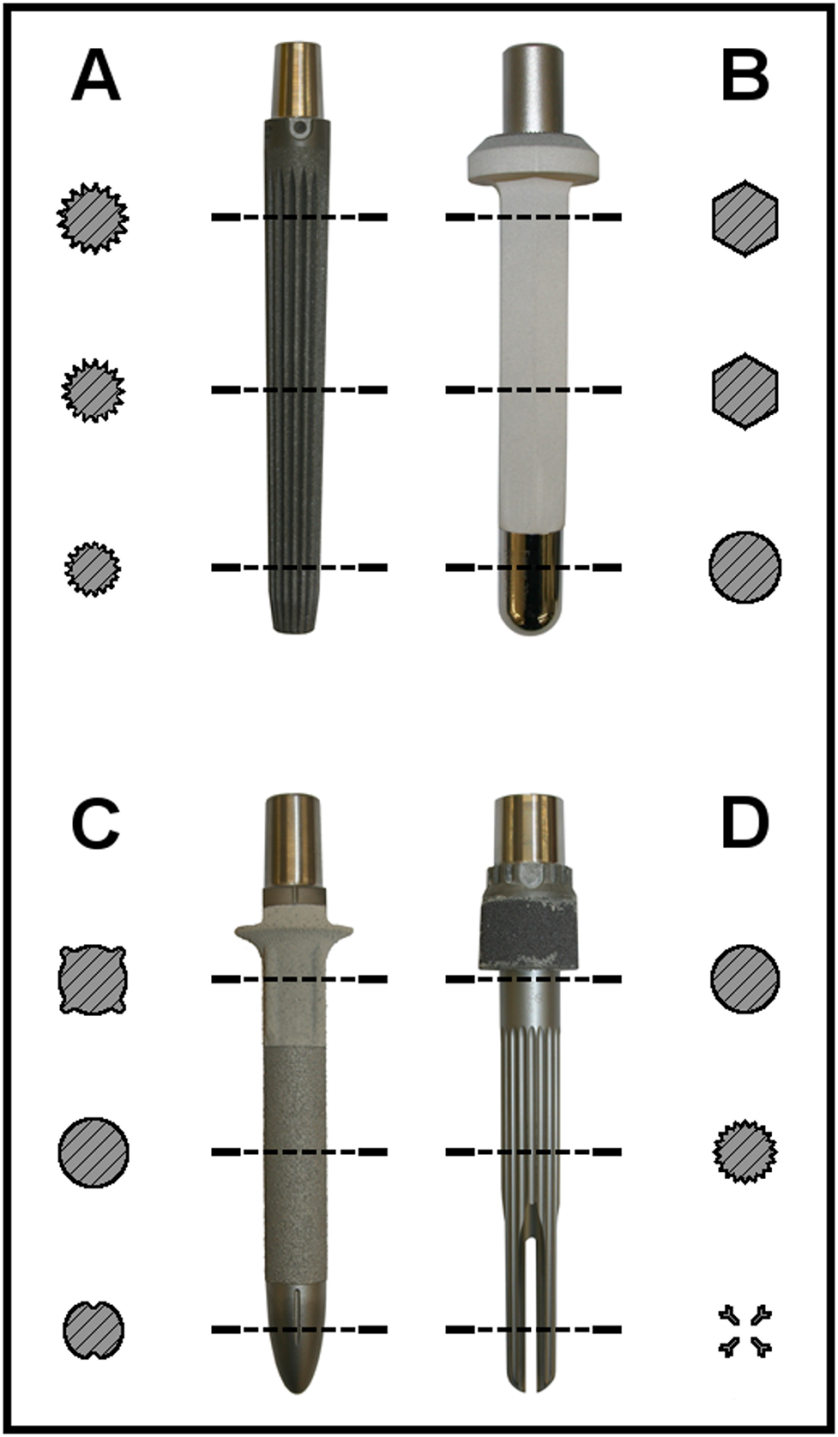
Schematic cross sections of all four stem designs in comparison.

Four cementless megaprostheses of the proximal femur are included in this study. Different geometries, surface modifications and design specifications (Fig 2) should offer different fixation methods. Some of the stems are available in multiple lengths. We chose comparable lengths to focus on the effect of bone defect size and remaining implant-bone contact area on the implant stability. The straight **Megasystem-C** (Group A: size 18, length 130 mm, LINK GmbH, Hamburg, Germany) stem has a conical design with a narrowed distal tip. The stem is fluted and porous-coated over its full length. The curved Modular Universal Tumor And Revision System **MUTARS** (Group B: size 17, length 120 mm, Implantcast GmbH, Buxtehude, Germany) stem has a hexagonal cross-section and a hydroxyapatite coating. In contrast to the rest of the stem, the design of the distal tip is cylindrical with a polished surface. The straight stem of the Global Modular Replacement System **GMRS** (Group C: size 17, length 125 mm, Stryker Orthopaedics, Mahwah, USA) can be divided into three sections. Proximally, four longitudinal expansions can be found along the cylindrical stem. The porous-coated surface of the stem is proximally coated with hydroxyapatite. In contrast, there is no surface modification at the distally narrowed taper. The **Segmental System** (Group D: size 18, length 130 mm, Zimmer Inc., Warsaw, USA) stem is made of a CoCrMo-alloy instead of the TiAl_6_V_4_-alloy of the other compared stems. Proximally, a Trabecular Metal sleeve is attached followed by a cylindrical part of the stem without surface or design modifications. Further distal sharp fluted fins are completed by a double-slotted end of the stem.

In a previous study we analyzed primary stability of these four megaprostheses in an AAOS II bone defect with preserved isthmus [8]. In accordance to this study, we prepared sixteen synthetic femora (composite bone 4^th^ generation (#3406), Sawbones Europe, Malmö, Sweden) with a standardized segmental AAOS III bone defect [9] to study the influence of bone defect extension. Therefore the implant supporting femoral isthmus was partially resected by an osteotomy 11 cm distal to the lesser trochanter (Fig 1). A group size of *n* = 4 was shown to be sufficient based on statistical power analysis within a similar experimental set-up [8]; therefore, four bones were allocated to each implant group. An experienced surgeon carried out rasping of the bone and evaluated the subsequently performed X-ray control. A standardized protocol [8] was used to obtain comparable conditions; however, bone bed preparation of the prostheses was carried out according to each manufacturer’s instructions. Stems were implanted in a standardized manner using a material testing device. For this, a setting of 2 kN followed by 4 kN applying 25 cycles each was used, simulating intraoperative impaction forces of the surgeon and the first careful post-op loadings of the patient himself. A second X-ray controlled the final implant fit within the synthetic bone.

**Fig 3:**
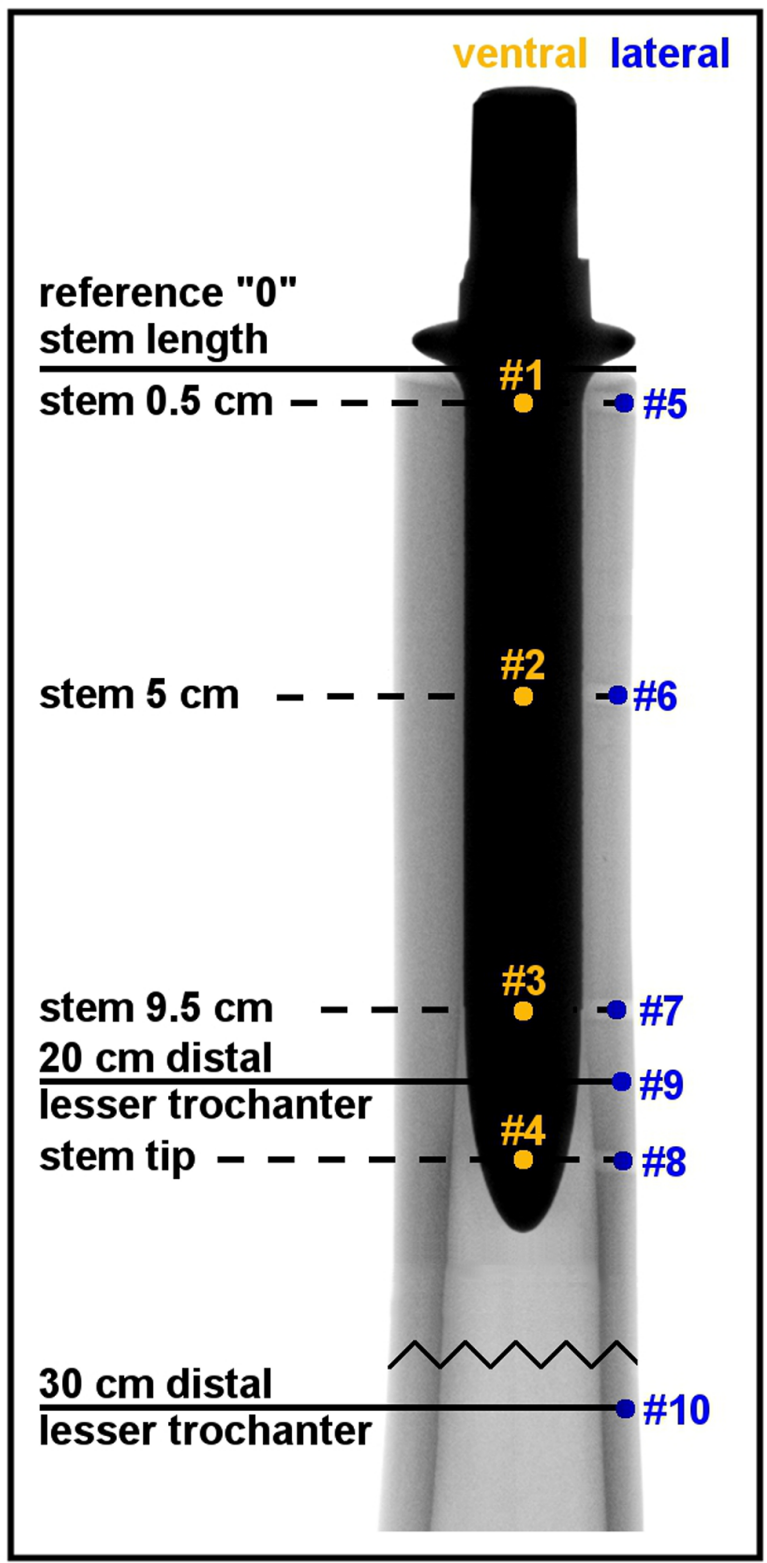
Measurement points of the implant (orange; #1-#4) and bone (blue; #5-#10) at different measuring levels.

The bones were fixed distally. Using two actuators with weights and a rope system, a cyclic torque of ±7 Nm was applied at the proximal end of the implant around its longitudinal axis. Subsequently, three-dimensional micromotions of the implant (Fig 3: measuring points #1-#4) and of the bone (Fig 3: measuring points #5-#10) were measured with six LVDT’s arranged in a 3-2-1-setup at each measurement point. Relative micromotions at the implant-bone-interface could be calculated based on a comparison of these absolute micromotions at each measuring level. The distribution of these relative micromotions along the longitudinal stem axis allows for the determination of the area of main fixation for each implant, as low relative micromotions correspond with tight fixation. The whole method of implant stability characterization has been described precisely and validated in our previous studies [8, 10–13].

**Fig 4:**
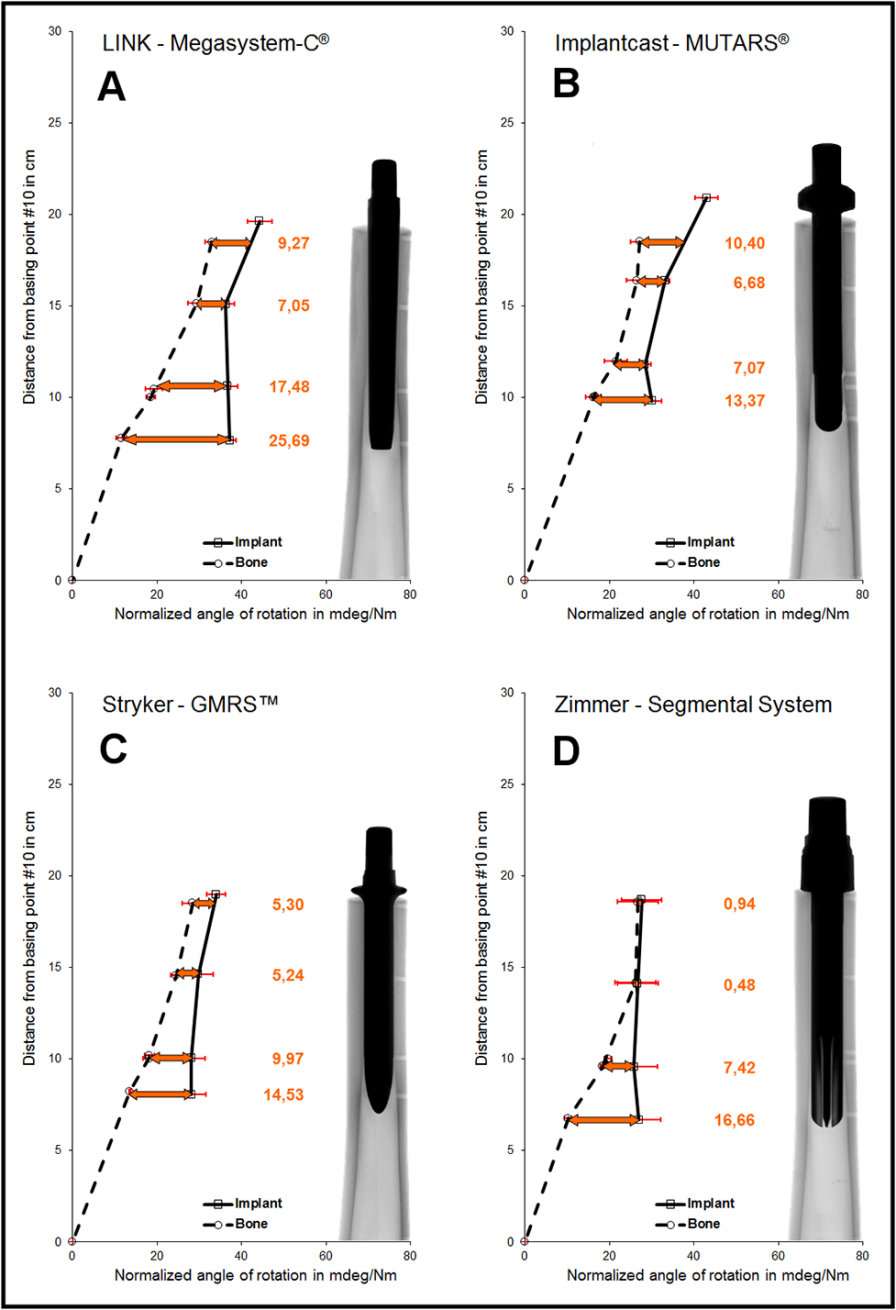
Rotational stability of a) Megasystem-C, b) MUTARS, c) GMRS and d) Segmental System in mdeg/Nm.

The characteristic fixation behavior in the presence of a proximal femoral isthmus resecting AAOS type III defect of all different stems are displayed in Fig 4. Each graph reflects the measured micromotions in mdeg/Nm of the femur (dotted line) and the implant (continuous line). Relative motions at the implant-bone interface are marked in orange. The X-rays of each implant show further information about the contact areas of bone and implant.

**Fig 5:**
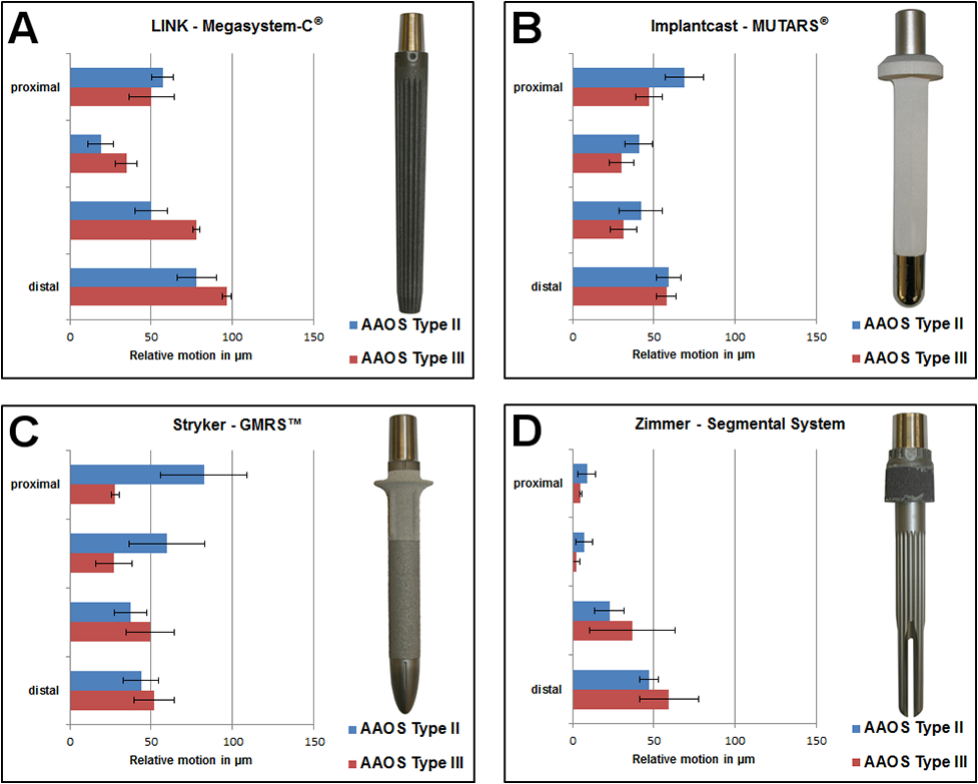
Converted relative micromotions in extreme case of stumbling [14] of a) Megasystem-C, b) MUTARS, c) GMRS and d) Segmental System in μm compared to AAOS defect type II [8].

Extent of relative micromotions is displayed in Fig 5. The measured micromotions in mdeg/Nm were converted to μm and extrapolated to an extreme case of joint loading (35 Nm in case of stumbling [14]). Loadings have been performed with downscaled loads to allow a comparable and non-destructive measurement; however, a linear correlation of motion and loading was shown [11]. All implant designs are separately displayed and compared directly to the smaller AAOS type II defects as previously published [8].

Statistics:A priori sample size estimation (α error = 0.05; decisive power of 0.80) was performed based on the results of a similar experimental set-up [8]. Using a statistic software (G*Power version 3.1.9.2, University Duesseldorf, Germany), a maximum group size of *n* = 4 was determined to be sufficient to characterize fixation characteristics of a single stem as well as to compare different implant groups using ANOVA. In addition, post hoc power analyses (α error = 0.05; groups = 4; measurements = 4) with ANOVA were performed for each measured implant group. Here, a power of 98–100% was achieved for each group, although the highest standard deviation within the groups was chosen as standard deviation parameter for each group within calculation of power. Analyses of variance were used to characterize the fixation of each megaprosthesis and to compare the fixation characteristics of all different implant designs. The results of this study were compared to those from our previous study [8] with an analysis of variance and Student’s T-tests to identify an influence of remaining femoral isthmus length on implant fixation. A “least significant difference” test (LSD) was used as a post-hoc test. A p-value <0.05 was considered to be significant. Data were expressed as means ± SD (standard deviation).

## Results

Within Group A (**Megasystem-C**), the lowest relative micromotions were measured in the proximal part of the stem near the proximal isthmus (Table 1; ~#1: 9.27±2.59 mdeg/Nm vs. ~#2: 7.05±1.34 mdeg/Nm; *p* = 0.06). The highest relative micromotions were located further distal at the distal end of the stem (~#4: 25.69±0.73 mdeg/Nm). Both proximal measuring points differ significantly compared to the points more distally located (each comparison *p*<0.01). The proximally significant lower micromotions indicate a proximal fixation characteristic in the extended AAOS type III defect. This implant fixation characteristic shows no significant difference to the smaller bone defect [8] (*p* = 0.06).

**Table 1:** Relative micromotions of all implant groups (A-D) and measurement points (from proximal to distal) described as means (Ø) and standard deviations (SD).

		Relative micromotion in mdeg/Nm			
Group		proximal	prox. isthmus	dist. isthmus	distal
**A**	**Ø**	9.27	7.05	17.48	25.69
	*±SD*	*2*.*59*	*1*.*34*	*0*.*47*	*0*.*73*
**B**	**Ø**	10.40	6.68	7.07	13.37
	*±SD*	*1*.*79*	*1*.*61*	*1*.*83*	*1*.*43*
**C**	**Ø**	5.30	5.24	9.97	14.53
	*±SD*	*0*.*47*	*2*.*19*	*2*.*99*	*3*.*46*
**D**	**Ø**	0.94	0.48	7.42	16.66
	*±SD*	*0*.*13*	*0*.*38*	*5*.*28*	*5*.*07*

Group A: Megasystem-C; Group B: MUTARS; Group C: GMRS; Group D: Segmental System.

Within Group B (**MUTARS**), the lowest relative micromotions were measured in the middle part of the stem near the isthmus (~#2: 6.68±1.61 mdeg/Nm vs. ~#3: 7.07±1.83 mdeg/Nm; *p* = 0.74). The highest relative micromotions were located further distally at the distal end of the stem (~#4: 13.37±1.43 mdeg/Nm). Both isthmus measuring points differ significantly compared to the distal point (each comparison *p*<0.01) and to the proximal measuring point (~#2: *p*<0.01 and ~#3: *p =* 0.02). The measured micromotions indicate a total or isthmus fixation characteristic in the extended AAOS type III defect. This overall implant fixation characteristic shows no significant difference to the smaller bone defect [8] (*p* = 0.14).

Within Group C (**GMRS**) lowest relative micromotions were measured in the proximal part of the stem near the proximal isthmus (~#1: 5.30±0.47 mdeg/Nm vs. ~#2: 5.24±2.19 mdeg/Nm; *p* = 0.97). The highest relative micromotions were located further distally at the distal end of the stem (~#4: 14.53±3.46 mdeg/Nm). Both proximal measuring points differ significantly compared to the distal point (each comparison *p*<0.01). The proximally significant lower micromotions indicate a proximal fixation characteristic in the extended AAOS type III defect. This implant fixation characteristic differs compared to the smaller bone defect (Fig 5)(*p* = 0.03), in which a distal fixation characteristic was determined [8].

Within Group D (**Segmental System**), the lowest relative micromotions were measured in the proximal part of the stem near the proximal isthmus (~#1: 0.94±0.13 mdeg/Nm vs. ~#2: 0.48±0.38 mdeg/Nm; *p* = 0.86). The highest relative micromotions were located further distal at the distal end of the stem (~#4: 16.66±5.07 mdeg/Nm). Both proximal measuring points differ significantly compared to the point more distally located (each comparison *p*<0.01). The proximally significant lower micromotions indicate a proximal fixation characteristic in the enlarged AAOS type III defect. This implant fixation characteristic is comparable to the smaller bone defect [8] (*p* = 0.55).

Apart from the fixation characteristics, the magnitude of relative micromotions at the different measuring levels vary between both bone defects in Group A, B and C (Fig 5). Except for the proximal part of the stem, the relative micromotions in Group A increased with bone defect extension, even significantly in the middle part of the implant (AAOS type II vs. AAOS type III; ~#2: *p* = 0.02 and ~#3: *p* = 0.01). In Group B all relative micromotions slightly decreased with widened bone resection, especially at the proximal part of the stem (AAOS type II vs. AAOS type III; *p* = 0.02); however, the overall fixation characteristic did not change with increased defect size. The proximal relative micromotions in Group C decreased significantly with bone defect extension (AAOS type II vs. AAOS type III; ~#1: *p* = 0.03 and ~#2: *p<*0.05) and increased distally. In contrast to Groups A-C, extent of relative micromotions at the different measuring levels between both bone defects was comparable in Group D. However, a tendency of decreasingly proximal micromotions and increasingly distal micromotions could be observed in the AAOS type III defect.

## Discussion

Aseptic loosening of megaprostheses is a relevant clinical problem [2, 4, 15, 16], especially considering the mostly young patients undergoing resection of bone tumors and reconstruction of the resulting defect with such implants.

The extent of required bone resection determines the fixation conditions. If the femoral isthmus remains intact, reliable cementless fixation of the implant can be achieved in this bone section with strong cortices [8]. If more of the proximal femur has to be resected, it can be suspected that the fixation might be complicated, as only the distal part of isthmus and widening cortical bone below are available (Fig 1). The AAOS defect classification [9], which considers mainly preserved bone for press-fit implantation, is commonly used [17], well reproducible, and the resulting defects correspond to resections in orthopaedic oncology. We therefore applied this classification to create standardized segmental defects. In a previous study [8] primary stability of megaprostheses in bones with maintained isthmus were studied. To analyze how altered fixation conditions influence the primary implant stability, we extended the femoral defect according to an AAOS type III defect with partial resection of the femoral isthmus.

Adapting the obtained results to physiological hip joint loadings according to Bergmann [14] all implants showed relative micromotions below 150 μm between bone and implant. Therefore, all implants might provide sufficient primary stability in an AAOS type III defect to allow bone integration [6, 7, 18]. However, maximum appearing relative micromotions in Group A and Group D increased from small AAOS type II defect [8] (A: 78.17±12.10 μm and D: 47.21±5.89 μm) to an extended AAOS type III defect (A: 96.61±2.75 μm and D: 59.47±18.11 μm). In contrast, maximum relative micromotions in Group B and Group C decreased with widened defect situation (small defect[8]: B: 68.82±11.53 μm and C: 82.27±26.66 μm vs. extended defect: B: 57.73±6.20 μm and C: 51.88±12.36 μm).

The **Megasystem-C** (Group A) exhibited a proximal fixation behavior with significantly less relative micromotions at both proximal when compared to both distal measurement points (Fig 4). The fixation was comparable to the AAOS type II defect [8] but a tendency toward a more homogeneous fixation pattern at the proximal stem was observed (Fig 5). The different shape of bone can explain the subtle change in fixation pattern, even though the stems can be characterized as proximal fixation in both defect types. The AAOS type II defect ends approximately 1cm above the isthmus. Therefore the most proximal part of the stem might not have been in close contact to the bone, as this narrows at this level to form the isthmus below (Fig 1).

It could be assumed, that the forces consequently could be more equally distributed in the AAOS type III defect and even though the fixation pattern still consists in locking in the narrowest part of the femur, peak forces with the associated risk of fracturing might be reduced as indicated by the significant change of relative micromotions at the proximal isthmus (Figs 4 and 5).

Due to the widening bone below the isthmus and the conical stem design (Fig 2), the relative micromotions at the distal part of the implant increase, which was significant at the distal isthmus (Fig 4).

For the **MUTARS** system (Group B), a homogenous fixation pattern in the isthmus could be demonstrated with significantly less primary stability at the proximal and distal stem parts (Fig 4). This hasn’t changed compared to the AAOS type II defect and can be explained by the stem design. It achieves stability with the cylindrical and curved shape (Fig 2) according to the distance with cortical contact. Apart from the very proximal part of the stem, no significant differences in respect to the extent of relative micromotions could be observed. The significantly increased primary stability proximally could be attributed to closer contact between the proximal stem and the isthmus in the AAOS type III defect, as already discussed for the **Megasystem-C**.

A proximal fixation pattern of the **GMRS** (Group C) could be established with significantly lower micromotions proximally and at the proximal isthmus compared to the distal measurement points. Considering the distally accentuated fixation in the AAOS type II defect [8], a significant change in fixation pattern was detected. As expected for implants with distal fixation, this change has to be attributed to the varied fixation conditions, as the distal part of the stem does not engage in the widening cortices below the isthmus to the same extent as within the isthmus. Surprisingly, the change considering the extent of micromotions is more pronounced at the proximal stem and proximal isthmus, where the reductions reach statistical significance (Fig 5). Furthermore the maximum relative micromotions of the whole stem were lower in the AAOS type III compared to type II defect [8]. As discussed earlier, a reduction in micromotions very proximally could be explained by the direct contact of the stem to the bone in the isthmus, which might have been prevented by the widening bone above the isthmus in AAOS type II defect. Considering the cylindrical design of the **GMRS**, we initially did not expect a distally accentuated fixation but a more homogenous fixation as observed with the **MUTARS**. In contrast to the latter, the **GMRS** stem is straight (Fig 2) and therefore the tip of the stem might have been in contact with the cortex of the bowed femur, resulting in less micromotions distally. In this case, a further subsidence of the stem and therefore engagement of the proximal expansions with the bone might have been avoided in the AAOS type II defect. With the widening cortices below the isthmus, a jamming of the stem tip might be prevented, which would allow optimal stem insertion and could explain the proximally significantly reduced micromotions as a result of the now effective proximal expansions.

The **Segmental system** (Group D) shows a proximal fixation pattern with significantly higher primary stability of the proximal measuring points, which has not changed by variation of the bone defect. Compared to AAOS type II defect no significant changes of relative micromotions could be observed, although a slight increase in relative micromotions at the distal stem could be explained by the impaired fixation conditions below the isthmus.

As we are simulating clinical situations, limitations of this study have to be considered. The experimental setup used is limited due to several factors; however, as earlier studies with similar methods could be confirmed clinically [10,11], we are convinced that our methods reflects a highly scientific standard. Applying a pure axial torque did not reflect all forces occurring in-vivo (e.g. within walking, stair climbing, etc.) [19]; nevertheless the torque chosen represents a high relevance for stem loosening in case of distortion. As a benefit compared to other studies, the axial torque application with ropes allowed to measure motions free of guided counteractions. The decreased torque of 7 Nm compared to other studies showing more realistic in-vivo loads [19] is another limitation. A decreased torque was used to allow a non-destructive measurement and to correspond to an expected moderate post-operative loading in case of such large defect situations. A linear correlation between force and motion could be confirmed in the past [11]. The non-destructive measurement is essential for consecutive motion analysis with LVDT’s at multiple measuring points, which also is a limiting factor.

Besides the level of resection, which we chose according to the clinically relevant AAOS classification [17], bone defects can also affect the integrity of the cortical bone. In this study the cortices of the sawbones were intact, which might not reflect the situation during a revision surgery after aseptic loosening of total hip arthroplasty but should correspond to oncologic resections in healthy bone of young patients.

Moreover, the shape and dimensions of the proximal femur can be variable between patients [20, 21], for which we did not account by using standardized sawbones in order to obtain reliable measurements. Although the femoral canal is prepared according to the shape of the implanted stem, our results might not be applicable to femora with a substantially different morphology compared to the tested sawbones in which the shape of the isthmus was characterized by parallel cortices over its entire distance (Fig 1).

For these sawbones mechanical characteristics could be demonstrated, which were consistent with cadaveric femurs at a lower variability [22, 23]. Nevertheless deviations of the sawbone properties from human bone properties can influence the validity of the obtained results. In addition, the normalized bone quality of the Sawbone might differ to the bone quality of a patient after a long and drug intensive therapy. With the applied study protocol and identical sawbones, however, clinically sound results in primary and revision surgery could be demonstrated in earlier studies [10, 11].

Megaprostheses with different designs were purposely chosen to study the effect of varied fixation conditions on their primary stability. Unfortunately their length varied slightly and one of the stems is only available in a curved form. Besides their shape and surfaces, these factors might also influence the different performance of stems and therefore limit the significance of our results. Biological effects cannot be studied in this biomechanical model, and as a result, the effect of hydroxyapatite coating or Trabecular Metal sleeves, which presumably will enhance bone integration [24, 25], cannot be assessed. The same is true for other factors leading to aseptic loosening, for which primary stability is only a prerequisite. This might also explain relevant clinical aseptic loosening rates, although we did establish an adequate implant fixation in our study.

In conclusion, we could demonstrate in this setting after partial resection of the femoral isthmus probably sufficient primary stability of all implants to allow bone integration and hence possible achievement of secondary stability to avoid aseptic loosening. After extension of the bone defect for most implants no significant change in fixation pattern was observed as long as a portion of the femoral isthmus was preserved for press-fit fixation. For the cylindrical stem with previously distal fixation, a significant conversion to a proximal fixation pattern was observed with an increase of its overall primary stability. Due to widening cortices below the isthmus in the AAOS type III bone defect most implants showed a tendency toward increased relative micromotions at the tip of the stem.

For clinical application the results of this study implicate that all analyzed megaprostheses provide adequate primary stability if the isthmus is at least partially preserved. The potential consequences of different force transmissions of implant and bone as stress shielding and thigh pain [18] are beyond the scope of this study. Nevertheless, the consideration of these aspects could influence surgeons in their choice of implants.

As we could not provide evidence to favor one of the studied implants in terms of superior primary stability, we can accordingly not recommend the application of a specific implant. However, our results were obtained in a sawbone model in which the femoral morphology was characterized by an isthmus with parallel cortices over a longer distance. The fixation conditions might be different in femora with a shorter, more converging, and narrower isthmus in which conical stems might be more appropriate.

Therefore the variability in the dimensions and shapes of the proximal femur [20, 21] should be considered to choose the implant which best provides tight diaphyseal contact over a long distance in the femoral isthmus to reconstruct extended proximal femoral bone defects.
